# A case–control study to evaluate hematological indices in blood of diabetic and non-diabetic individuals in Ibb City, Yemen

**DOI:** 10.1038/s41598-023-43973-3

**Published:** 2023-10-04

**Authors:** Abdul Baset Abbas, Alia Hazeb, Rawan Al-Badani, Boshra Al-Thmary, Rasha Mokaram, Somayah Al-Najjar, Shifa Mothna, Aziza Kssiam, Abeer Esmail, Ali Al-Rashidi

**Affiliations:** 1 Medical Laboratories Department, Faculty of Medicine and Health Sciences, Ibb University, Ibb City, Yemen; 2 Department of Medical Laboratories, Faculty of Medical Sciences, Aljazeera University, Ibb City, Yemen; 3 Department of Medical Microbiology, Faculty of Sciences, Ibb University, Ibb City, Yemen; 4 Zain Medical Laboratories, Ibb City, Yemen

**Keywords:** Biochemistry, Biological techniques, Cell biology, Immunology, Microbiology, Physiology

## Abstract

Diabetes mellitus (DM) is a chronic, metabolic illness characterized by an elevation of blood sugar levels. Patients with diabetes show changes in hematological indices. The study aimed to determine hematological indices, ESR, CRP, blood pressure (BP), and weight and their relationship with a fasting blood sugar (FBS) level and different variables in diabetic mellitus patients (DM) compared with healthy control (HC). A total of 202 participants (102 DM group and 100 HC group) were selected randomly. Data were collected using a questionnaire. Blood samples were collected from different places and investigated in Zain Medical Laboratories in Ibb City, Yemen (September 2022 to May 2023). GraphPad Prim was used to analyze the results. P-value ≤ 0.05 was considered statistically significant. The mean and standard deviation of age, weight, gender, residence, marital status, education levels, economic status, regular exercise, following a strict diet, and family history of diabetes revealed significant differences between DM and HC groups (P < 0.0001, P = 0001, P = 0.0027, P = 0.0002, P < 0.0001, P < 0.0001, P = 0.0002, P = 0.0011, P < 0.0001 and P = 0.0001, respectively). FBS results, systolic and diastolic BP, MCV, WBCs, monocytes, eosinophils, and platelets displayed significant differences between both groups (P < 0.0001, P < 0.0001 and P = 0.0404, P = 0.0191, P < 0.0001, P = 0.0253, P < 0.0001, and P = 0.0229, respectively). ESR exhibited statistical significance (P < 0.0001), while CRP displayed no significance. A Pearson's correlation showed that weight, Hb, RBCs, PCV, and WBCs were statistically negatively correlated with FBS whereas other hematological indices showed no correlation with FBS. In conclusion, DM patients had relatively higher levels of MCV, WBCs, eosinophils, platelets and ESR than the control group.

## Introduction

Diabetes mellitus (DM) is a chronic, metabolic illness characterized by an elevation of blood sugar levels, which leads to severe injury and dysfunction to heart, kidneys, blood vessels, eyes, and nerves^1–3^. The main types of DM are two; Type 1 diabetes mellitus, which has autoimmune origins, as a result of the destruction of β-cells in the pancreas, leading to defects in insulin production and Type 2 diabetes mellitus, which occurs in adults as a result of insulin resistance or inadequate production. Both types lead to a decrease or prevention of insulin production, which result in a group of metabolic imbalances accompanied by multiple disorders in the metabolism of lipid, carbohydrate, and protein^1,4–6^. DM is accompanied by hyperglycemia, hyperlipidemia, and glycosuria. Hyperglycemia is responsible for multiple physiological defects such as vasodilation, inflammatory regulation, immunological indices, hematological indices (size, morphology, and function of WBCs, RBCs, and platelets), and cell growth in uncontrolled diabetes^7,8^. Uncontrolled diabetes is responsible for cardiovascular diseases, nephropathy, neuropathy, lower-limb amputation, and retinopathy causing loss of vision and may be blindness^4,5,9,10^.

DM is a worldwide distribution where the total number of diabetes prevalence is 537 million individuals and approximately 6.7 million die annually which represent the third rank after cardiovascular disease and malignant tumor^9^. Additionally, there are many studies conducted in different countries in the Middle East indicating that the prevalence of diabetes in Yemen is the lowest^11–26^. It is noticeable that the prevalence rate increased from 2004 (4.6%) to 2019 (9.8%)^25,27–29^. In the blood of DM, the elevation of HbA1c and slow glycosylation can be associated with structural and functional variations in RBCs osmotic turbulences and cytoplasmic fluidity as well as hemoglobin molecules. These variations may be reflected in hematological indices such as RBCs count, Hb, mean corpuscular hemoglobin (MCH), mean corpuscular volume (MCV), and mean corpuscular hemoglobin concentration (MCHC)^30–35^. There are many studies conducted in many countries to study the changes in hematological indices, blood pressure (BP), weight, ESR, and CRP and their relation with diabetes^4,35–39^. There is no previous study on hematological indices in Yemen. The current study aimed to detect hematological indices, ESR, CRP, weight, and BP and their relationship with FBS and sociodemographic, clinical, and anthropometric variables in DM group compared with HC group.

## Methods and materials

A case–control study was conducted in Ibb City et al. Thawrah Hospital, Majd Specialist Hospital, Ibb Medical Center, Al-Kamal Laboratories, Al-Rafah Laboratories, and Zain Medical Laboratories from September 2022 to May 2023. Ibb City is distant from Sana'a, Yemen's capital, about 193 km.

The study participants were selected randomly. To detect the sample size, we followed the thumb rule, suggested by van Voorhis and Morgan, in that at least 30 participants per group are required to detect the real differences that lead to about 80% power of the study if the study contains two or more groups, as well as at least 50 participants per group, are required to detect the correlation relationship^40^. To maximize the reliability of the study, the researchers tried to increase the number of study participants, as suggested by the thumb rule, where 100 participants were required in each group (100 DM and 100 healthy controls). Moreover, for both DM and HC groups, the participants were screened for FBS, and healthy people in a control group who had a high level of sugar; were counted as DM (2 cases). So, the study included 202 participants involving 102 DM group and 100 HC group. Furthermore, the participants were interviewed using a standardized predesigned questionnaire. The questionnaire was translated into Arabic, the Yemen's mother tongue. The data collected included the participant number, weight, age, gender, residence, marital status, education status, socioeconomic status, type of diabetes, family history of diabetes, smoking, qat chewing, tobacco chewing, etc. Approximately, 3ml of blood was collected with EDTA-K3, and 1.28 ml of blood was drawn into a tube containing 0.32 ml 3.8% sodium citrate 4NC. Blood samples were collected from the participants after overnight fasting. The whole blood was tested for completing blood count (CBC) and erythrocyte sedimentation rate (ESR) whereas the serum was used to detect fasting blood sugar (FBS) and C-reactive protein (CRP) CRP. All tests were done in Zain Medical Laboratories (ZML). Additionally, blood pressure (BP) level was measured.

Concerning the inclusion criteria for both DM and HC groups, the participants who were ≥ 16 years of age were included. Moreover, the participants (DM or HC groups) who had any history of chronic liver disease including hepatitis B virus (HBV) and hepatitis C virus (HCV), heart failure, renal disease, malignancy, bleeding disorders, infectious diseases, and pregnant women were excluded. Also, the participants HC group who suffered from tonsillitis, arthritis, and appendicitis were excluded.

### Research questions

Based on the previous studies, this study attends to address the following research questions:

What are the hematological indices of healthy people?

What are the hematological indices of diabetes mellitus patients?

Are there any statistically significant differences between healthy and DP patients?

### Ethical considerations

The ethical clearance of this study was approved by the Medical Laboratories Department (MDL), Faculty of Medical Sciences, Al-Jazeera University ethical committee after due process was followed (Reference Number: MDLMSJU/0101/2022 on 15 August 2022). The Ethics Committee accorded with the Helsinki Declaration for the Protection of Human Subjects. Informed consent was obtained from all the study participants.

### Data analysis

The data obtained from the results of the questionnaires and laboratory tests were evaluated using GraphPad Prism 7.01 (GraphPad Inc. USA). A Kolmogrov-Smirnov test (KST) was used to check the variables' distributions. A Mann–Whitney test was used for a non-normally distributed date and an unpaired (independent) *t*-test was used for a normally distributed date. Sociodemographic and anthropometric characters were analyzed with a chi-square test for trend or Fisher exact test. Additionally, a Pearson's correlation (PC) was used to display the association relation of FBS with hematological indices. The results presented percentages (%) and P values. P values ≤ 0.05 were considered statistically significant.

## Results

### Sociodemographic characteristics of study participants

In this investigation, 102 participants were DM and 100 were enrolled as non-diabetic as a HC group. All variables were statistically analyzed including mean, standard deviation (SD) and range as shown in the tables. The mean of age, and SD were 48.61 ± 14.78 years for the DM group and 31.7 ± 10.36 years for the HC group. Moreover, the mean and SD of weight were 65.11 ± 14.33 Kgs for DM group and 57.42 ± 9.756 Kgs for HC group. In DM group, 52 (50.98%) were females and 50 (49.02%) were males while among HC group 70 (70%) were females and 30 (30%) were males. Additionally, about 80 (78.43%) and 96 (96%) of study participants were included in DM and HC groups respectively were living in urban areas. The majority of the DM group was married 94 (92.16%), unemployed 71 (69.61%), and had the lowest education (illiterate) 40 (39.21%). Whereas the majority of the HC group was single 64 (64%), unemployed 68 (68%), and had the highest education (university) 70 (70%). Furthermore, 69 (67.65%) of DM group were middle economic level and 86 (86%) of HC group were middle economic level. Moreover, most of the participants in DM group were non-smokers, non-tobacco chewing, qat chewing, non-regular exercise, and non-follow a strict diet with the following number and percentages respectively 85 (83.33%), 97 (95.10%), 67 (65.69%), 70 (68.63%) and 57 (55.88%). Likewise, most participants in HC group were non-smokers, non-tobacco chewing, qat chewing, non-regular exercise, and non-follow a strict diet with the number and percentage 88 (88%), 100 (100%), 52 (52%), 88 (88%) and 92 (92%), respectively. In addition, 64 (62.75%) and 32 (32%) of the DM group and HC group had a family history of diabetes, respectively as shown in Table 1.

**Table 1:** Sociodemographic characteristics of study participants.

Variable	Diabetic mellitus (DM) patients (102)	Healthy control (100)	*P-*value
Age (years) Mean ± SD	48.61 ± 14.78 (16 ~ 80)	31.7 ± 10.36 (20 ~ 72)	< 0.0001
Weight (Kgs) Mean ± SD	65.11 ± 14.33 (39 ~ 105)	57.42 ± 9.707 (32 ~ 94)	0.0001
Gender			
Male	50 (49.02%)	30(30%)	0.0027
Female	52 (50.98%)	70 (70%)	
Residence			
Urban	80 (78.43%)	96 (96%)	0.0002
Rural	22 (21.57%)	4 (4%)	
Marital status			
Married	94 (92.16%)	36 (36%)	< 0.0001
Single	8 (7.84%)	64 (64%)	
Employment status			
Employed	31 (30.39%)	32 (32%)	0.8796
Unemployed	71 (69.61%)	68 (68%)	
Education levels			
Illiterate	40 (39.21%)	4 (4%)	< 0.0001
Primary	29 (28.43%)	14 (14%)	
Secondary	12 (11.76%)	12 (12%)	
University	21 (20.60%)	70 (70%)	
Economic status			
Low	5 (4.90%)	8 (8%)	0.0002
Middle	69 (67.65%)	86 (86%)	
High	28 (27.45%)	6 (6%)	
Smoking			
Smokers	17 (16.67%)	12(12%)	0.4234
Non smokers	85 (83.33%)	88 (88%)	
Tobacco chewing			
Yes	5 (4.90%)	0 (0%)	0.0595
No	97 (95.10%)	100 (100%)	
Qat chewing			
Yes	67 (65.69%)	52 (52%)	0.0628
No	35 (34.31%)	48 (48%)	
Regular exercise			
Yes	32 (31.37%)	12 (12%)	0.0011
No	70 (68.63%)	88 (88%)	
Follow a strict diet			
Yes	45(44.12%)	8(8%)	< 0.0001
No	57 (55.88%)	92 (92%)	
Family history of diabetes			
With	64 (62.75%)	32 (32%)	0.0001
Without	38 (37.25%)	68 (68%)	

In this study, as presented in Table 1, there was a statistically significant between DM and HC groups in age, weight, gender, residence, marital status, education levels, economic status, regular exercise, follow a strict diet, and family history of diabetes. On the other hand, there was no significance between DM group and HC group in employment status, smoking, tobacco chewing, and qat chewing.

### Clinical and anthropometric variables among diabetic mellitus (DM) patients

Briefly, a total of 102 DM group participants were included. Out of DM group participants, about 24 (23.53%) were suffering from Type 1 diabetic mellitus, 55 (53.92%) from Type 2, and 23 (22.55%) they do not know. Regarding the duration of diabetic disease, the mean of diabetic duration and SD were 6.489 ± 6.274 years as well as the most of DM group had a duration ≤ 5 years (61.76%). Furthermore, the treatment profile showed that 87 (85.29%) used diabetic treatment, out of 87 used diabetic treatment, 62 (71.26%) utilized oral tablets, and 25 (28.74) were injected insulin. Additionally, 60 (58.82%) had eye problems with 33/60 (55%) retinopathy, 26/60 (43.33%) cataracts, and 1/60 (1.67%) blindness. Furthermore, 8 (7.84%), 17 (16.67%), 40 (39.22%), 18 (17.65%), and 47 (46.08%) had renal failure, foot ulceration, gingivitis, heart disease, and numbness in feet or legs, respectively. Regarding the inflammatory symptoms, about 27 (26.47%), 61 (59.80%), and 4 (3.92%) had tonsillitis, arthritis, and appendicitis, respectively as revealed in Table 2.

**Table 2:** Clinical and anthropometric variables of diabetic mellitus (DM) patients.

Variables	Categories	Diabetic mellitus (DM) patients no. (%)
Type of diabetes	Type 1	24 (23.53%)
	Type 2	55 (53.92%)
	Don’t know	23 (22.55%)
Duration of diabetes	6.489 ± 6.274 (1 month ~ 30 years) Mean ± SD (range)	
	≤ 5 years	63 (61.76%)
	> 5 years	39 (38.24%)
Diabetes treatment usage	Yes	87 (85.29%)
	No	15 (14.71%)
Treatment regimen	Oral (tablets)	62/87 (71.26%)
	Injection (insulin)	25/87 (28.74)
Eye problem (60–58.82%)	Retinopathy	33/60 (55%)
	Cataracts	26/60 (43.33%)
	Blindness	1/60 (1.67%)
Renal failure	Yes	8 (7.84%)
	No	94 (92.16%)
Foot ulcer	Yes	17 (16.67%)
	No	85 (83.33%)
Gingivitis	Yes	40 (39.22%)
	No	62 (60.78%)
Heart disease	Yes	18 (17.65%)
	No	84 (82.35%)
Numbness in feet or legs	Yes	47 (46.08%)
	No	55 (53.92%)
Tonsillitis	Yes	27 (26,47%)
	No	75 (73.53%)
Arthritis	Yes	61 (59.80%)
	No	41 (40.20%)
Appendicitis	Yes	4 (3.92%)
	No	98 (96.08%)

### Comparison of hematological indices between diabetic mellitus (DM) patients group and healthy control group

In this work, as presented in Table 3, there were no statistically significant differences between the mean and SD of DM group and HC group respecting Hb level, RBCs count, PCV, MCH, MCHC, neutrophil count, lymphocyte count, and basophil count whereas the findings revealed that the mean and SD of FBS, systolic and diastolic BP, MCV, WBCs count, monocyte count, eosinophil count, platelets count, and 1 h and 2 h ESR had statistical significance between DM group and HC group. Out of the participants, about 53 (51.96%) of DM group were CRP positive, and about 40 (40%) of HC group were CRP positive, there was no significance between DM group and HC group in CRP assay results.

**Table 3:** Comparison of hematological indices between diabetic mellitus (DM) patients group and healthy control group.

Variable	Diabetic mellitus (DM) patients	Healthy control	*P*-value
	Mean ± SD (range)	Mean ± SD (range)	
FBS mg/dl	233.9 ± 66.7 (155–460)	95.9 ± 9.946 (77–119)	< 0.0001
Blood pressure (mmHg)			
Systolic	124.2 ± 20.5 (70 ~ 215)	110.1 ± 9.597 (90 ~ 130)	< 0.0001
Diastolic	79.65 ± 12.1 (50–100)	77.2 ± 8.05 (60–90)	0.0404
CRP assay			
Negative	49 (48.04%)	60 (60%)	0.0927
Positive	53 (51.96%)	40 (40%)	
Hb g/dl	14.21 ± 1.856 (8.9 ~ 18.5)	14.01 ± 1.625 (11.3 ~ 17.8)	0.4262
RBC(× 10^12^/l)	4.996 ± 0.6349 (2.67 ~ 6.08)	4.921 ± 0.4971 (4.19 ~ 6.48)	0.3537
PCV%	41.95 ± 5.326 (22 ~ 53.1)	41.24 ± 5.375 (24.1 ~ 53.1)	0.3454
MCV fl	84.47 ± 11.42 (40.4 ~ 170.3)	80.2 ± 14.19 (29.8 ~ 93.2)	0.0191
MCH pg	28.66 ± 4.589 (10.2 ~ 59.5)	28.31 ± 2.132 (18.7 ~ 31.7)	0.4853
MCHC g/l	33.6 ± 1.348 (23.4 ~ 35.7)	33.5 ± 1.058 (28.4 ~ 35)	0.5585
WBCs (× 10^9^/l)	7.97 ± 2.649 (3.91 ~ 15.25)	4.901 ± 1.067 (2.73 ~ 7.1)	< 0.0001
Neutrophils%	47.87 ± 12.24 (20.2 ~ 80.8)	46.81 ± 7.338 (30 ~ 59.5)	0.4636
Lymphocytes%	40.44 ± 11.29 13 ~ 69	38.99 ± 10.14 (16.7 ~ 59.1)	0.3381
Monocytes%	7.335 ± 2.342 (0.5 ~ 13.5)	8.098 ± 2.467 (3.9 ~ 20.3)	0.0253
**Eosinophils%**	3.821 ± 2.946 (0.3 ~ 15.2)	2.218 ± 1.541 (0.3 ~ 6)	< 0.0001
**Basophils%**	0.4206 ± 0.2538 (0 ~ 1.1)	0.384 ± 0.2295 (0 ~ 1)	0.2841
**Platelets (× 10**^9^**/l)**	295.3 ± 84.06 (140 ~ 562)	272.9 ± 49.69 (115 ~ 350)	0.0229
**ESR 1 (mm/hr)**	35.31 ± 18.83 (10 ~ 90)	18.48 ± 8.813 (4 ~ 35)	< 0.0001
**ESR 2 (mm/hr)**	50.12 ± 22.12 ((19 ~ 111)	29.4 ± 11.22 (8 ~ 60)	< 0.0001

### Pearson's correlation coefficient (r) of hematological indices with FBS among study participants

Pearson's correlation coefficient (r) showed that weight, Hb levels, RBCs count, PCV, and WBCs count were statistically negatively correlated with FBS in DM group. Moreover, no statistically significant correlation coefficient noted between the FBS and other hematological indices of the DM group. Furthermore, in HC group, as demonstrated in Table 4, FBS and platelets exhibited a statistically positive correlation coefficient (r = 0.334; *P* = 0.0007), while the other hematological indices revealed no statistical correlation.

**Table 4:** Pearson's correlation coefficient (r) of hematological indices with FBS among diabetic group and control group.

Variable	Diabetic mellitus (DM) patients		healthy control	
	r	*P*-value	r	*P*-value
Age	0.1514	0.1287	− 0.1396	0.1661
Weight	− 0.2011	0.0427	0.1852	0.0651
Blood pressure				
Systolic	− 0.1332	0.1821	− 0.0101	0.9206
Diastolic	− 0.0255	0.7992	− 0.1082	0.2837
Hb g/dl	− 0.2762	0.0049	− 0.0123	0.9033
RBC(× 10^12^/l)	− 0.2577	0.0089	0.01216	0.9044
PCV%	− 0.3227	0.0009	− 0.02608	0.7967
MCV fl	− 0.02722	0.7859	0.078	0.4405
MCH pg	− 0.003397	0.9730	-0.0554	0.5840
MCHC g/l	− 0.0574	0.5666	0.0395	0.6964
WBCs (× 10^9^/l)	− 0.2175	0.0281	0.01835	0.8562
Neutrophils%	0.01036	0.9177	0.06581	0.5153
Lymphocytes%	− 0.005207	0.9586	0.08183	0.4183
Monocytes%	0.03827	0.7026	− 0.1919	0.0558
Eosinophils%	0.1093	0.3407	0.1265	0.2098
Basophils%	0.02466	0.8057	0.1675	0.0959
Platelets (× 10^9^/l)	− 0.002298	0.9817	0.334	0.0007
ESR 1 (mm/h)	0.006463	0.9486	− 0.05499	0.5868
ESR 2 (mm/h)	− 0.1177	0.2387	− 0.09284	0.3583

## Discussion

DM is a chronic illness associated with the elevation of blood glucose levels because the human body cannot produce enough insulin or efficiently employ insulin. Patients with DM showed an important derangement in various hematological indices^4,41^. This study involved a comparison of the FBS levels between HC group and DM group. We noted a significant variance in FBS levels between DM group and HC group (*P* < 0.0001). Additionally, the study involved a comparison of BP between both groups, where the comparison results showed significant differences among systolic and diastolic pressures with *P* values < 0.0001 and 0.0404, respectively. This result is in accordance with a study conducted in Gondar; Ethiopia^35^. This might be due to the direct and toxic effects of chronic hyperglycemia on endothelial cells of the vascular which lead to vascular repairs and increased vasoconstriction ultimately affecting BP^35,42^. Likewise, RBC indices had shown non-statistically significant. This result is in agreement with that described by various studies in Sudan, Iran, USA, and Taiwan^37,43–45^.

In this study, an Unpaired T-test displayed that the mean and SD of MCV, the total count of WBCs, eosinophil, and the count of platelet were highly statistical significant in DM group than HC group (*P* = 0.0191, *P* < 0.0001, *P* < 0.0001 and *P* = 0.0229, respectively). On the other hand, monocyte was statistically significantly low in DM group than in the HC group. However, the mean and SD of Hb level, RBC, PCV, MCH, MCHC, neutrophil, lymphocyte, and basophils in this study were not statistically significant. There are many studies conducted in different countries showing variations in hematologic indices, some studies showed significance, while others did not. The mean and SD of basophil, monocyte, RBCs, MCH, and MCV were statistically variant between DM and HC groups in Dessie and Gondar cities of Ethiopia, Bosnia and Herzegovina, India, Sudan, and Nigeria^4,35–37,46,47^. The findings report of monocyte and basophil in Nigeria were significantly low in DM as compared to HC group^47^ while other studies in Turkey, Ethiopia, and India in which monocyte was statistically high in DM group as compared to HC group^36,48,49^. The results of the mean of MCV and MCH in Brazil, Ethiopia, Saudi Arabia, India, and Sudan were statistically low in DM compared with HC group^30,50–53^. In dissimilarity, a study done in Saudi Arabia exhibited that MCHC was statistically high in DM group compared to HC group^52^.

In our investigation, the WBCs count was elevated in DM. This result was similar to studies conducted in Sudan and Taiwan^37,54^. Concerning both ESR results, after one or two hours were statistically significant. A previous study conducted in Iraq found that ESR was significantly higher^55^. This study also involved comparison of platelet between HC group and DM group. We noted significant variances in platelet between both groups. Regarding CRP assay result, there was no statistical significance. Our study was dissimilar to research carried out in India^38^. The variations might be attributed to dissimilarities in the size of the sample, geographical location, socioeconomic status, laboratory investigative technique used, and dissimilarities among the participants as well as diet, daily behavior, and daily practices of individuals^4^. Moreover, the present study exhibited that the weight, Hb level, RBCs, PCV, and total WBCs count were statistically negatively correlated with FBS in DM. This study did not align with studies done in Poland and Israel, where the levels of FBS proportionally increased for every raise in WBCs count^56,57^. PC between FBS and ESR showed a weak correlation with no relationship. Our results were similar to a study conducted in Pakistan^39^.

Our study concluded that many hematological indices can differentiate DM patients from HC individuals. DM patients had relatively higher levels of MCV, WBCs, eosinophils, platelets, and ESR than the control group. Further studies were recommended to complete what our study lacks such as identification of the relationship between HbA1C and hematological indices.

## Limitations

The study limits itself to Ibb City, Yemen which results in producing a small size. This occurs due to the absence of supporting institutions.

## Data Availability

The data sets generated during and/or analyzed during the current study are available from the corresponding author upon reasonable request.
